# A Descriptive Study of Some of the Dento-Gingival Components of Esthetics of Patients at a Tertiary Care-center in the Eastern Part of Nepal

**DOI:** 10.1155/2022/5589309

**Published:** 2022-10-22

**Authors:** Arati Sharma

**Affiliations:** Department of Prosthodontics, CODS, B.P. Koirala Institute of Health Sciences, Dharan, Nepal

## Abstract

**Background:**

The purpose of this study was to observe and describe some of the dento-gingival components of esthetics like the mean gingival zenith position (GZP) with respect to the vertical bisected midline axis (VBM), relative gingival zenith level (GZL) of lateral incisors (LIs), heights and widths of central incisors (CIs), LIs, and their ratios.

**Materials and Methods:**

This cross-sectional, hospital-based, descriptive study was conducted from Feb 2019 to Aug 2019. Ethical clearance was taken from the Institutional Review Committee of B. P. Koirala Institute of Health Sciences. Convenience sampling was done. Variables (above mentioned) were marked and measured on the casts with a calibrated digital vernier caliper and entered in the data sheet, and descriptive analysis was done with SPSS version 20.

**Results:**

A total of 210 subjects of the age group 18–25 years were included in the study. Mean GZP of the right CI was 0.49 ± 0.54 mm, of the left CI was 0.42 ± 0.43 mm, of the right LI was 0.20 ± 0.34 mm, of the left LI was 0.04 ± 0.35 mm, of the right C (Canine) was 0.023 ± 0.38 mm, and of left C was 0.07 ± 0.38 mm. Mean relative GZL of LI was about 0.40–0.41 mm below the reference line. Mean height of the right CI was 9.34 ± 0.93 mm, and that of the left CI was 9.31 ± 0.87 mm; that of the right LI was 7.91 ± 0.98 mm, and that of the left LI was 7.92 ± 0.91 mm. Mean width of the right CI was 8.34 ± 0.57 mm, and that of the left CI was 8.38 ± 0.48 mm; that of the right LI was 6.62 ± 0.58 mm, and that of the left LI was 6.66 ± 0.53 mm.

**Conclusions:**

Mean GZP of each upper anterior tooth was distally located with respect to VBM; that of CI was more distally placed than LI and C. Relative GZL of LI was below the reference line. The central incisor width/height ratio obtained was >80% that means a squarer tooth.

## 1. Introduction

Concern for esthetics is increasing in the general population. Some dental patients are so critical about their previous appearance and want the prosthetic tooth/restoration to be the same, whereas others want esthetic teeth irrespective of their previous appearance. Among the vital elements of smile design, tooth dimensions and zenith points are essential [1–3].

Regarding tooth dimensions, several guidelines have been proposed for creating the correct proportions of anterior teeth. Those guidelines are based on perceived proportions viewed from the facial aspect, and one of them is the golden proportion [4, 5]. The golden proportion is used to create a pleasing smile that is balanced with the face. The “rule of thirds” is applied while using this proportion, i.e., the CI (central incisor) should be 1.6 times as wide as the LI (lateral incisor), and C (canine) 0.6 times as wide as LI [6, 7]. Clinicians accept and apply this principle to dentistry, but, as it is a mathematical formula, it is rigid and has raised questions regarding its reliability [1].

The gingival margin of the CIs should match the Cs and should be slightly above the gingival margin of the LIs [8, 9]. The gingival zenith is the most apical aspect of the free gingival margin [4, 10, 11]. The location of the gingival zenith in a medial-lateral position relative to the vertical tooth axis of the maxillary anterior teeth is esthetically important. When there is a need to alter the mesial and distal dimensions of anterior teeth, one of the critical steps is to establish the proper location of zenith points [1].

Similar studies done in different populations are there in the literature [6, 8, 10–17]. At least one study done in Nepal but representing a different population is known to the author [18]. Still, there is insufficient reference/guide in the literature with respect to dento-gingival esthetics.

Thus, the purpose of this study was to measure and analyze some of the dento-gingival components of esthetics like gingival zenith position (GZP) with respect to the vertical bisected midline axis (VBM), relative gingival zenith level (GZL) of LIs, heights and widths of CIs, LIs, and Cs, and their ratios, which will be a guide or reference for the dentists to restore the previous appearance of teeth of such patients to a great extent and to communicate with the patient before any change in the appearance. Particularly, when a young to middle-aged patient comes to a dental clinic with an unexpected loss of anterior teeth or tooth material and there is no previous photograph/record for reference, it becomes quite challenging for the dentist to restore the natural look of anterior teeth. In such cases, this study will be a guide or reference to restore the previous appearance of the teeth to a great extent.

## 2. Materials and Methods

This cross-sectional, hospital-based, descriptive study was conducted from Feb 2019 to Aug 2019. Ethical clearance was taken from the Institutional Review Committee of B.P. Koirala Institute of Health Sciences (code no. IRC/1492/019). Informed written consent was taken from each participant to get enrolled in the study.

Convenience sampling was done. All patients who fulfilled the inclusion criteria during the study period were included. Inclusion criteria were all dentate and partially dentate patients with intact, well-aligned anterior teeth, and healthy periodontium of age group 18–25 years reported to the Department of Prosthodontics during the study period. The exclusion criteria for the patients were gingival and periodontal disease, spacing, crowding, rotation, severe proclination/retroclination, incisal attrition, restoration in upper anterior teeth, obvious facial deformities, history of disease that may alter the craniofacial morphology, history of maxillofacial, plastic or reconstructive surgery, and history of trauma involving anterior teeth.

A clinical examination was done, and an alginate impression of the upper arch was taken. A digital vernier caliper (Mitutoyo's Absolute Solar Digimatic Caliper) was used to measure the variables (GZP, GZL, and crown dimensions) on the casts obtained from alginate impressions of the upper arches, measurements were entered in a data sheet, and descriptive analysis was done with SPSS version 20. Variables were marked and measured following a standard method [11]. GZP dimensions were measured for each individual tooth in a medial-lateral direction from the VBM. GZLs were measured in an apical coronal direction from a tangent line drawn on the casts to the GZPs of the adjacent teeth. Heights of the upper incisors were measured along the VBM of the clinical crown. Widths of the upper incisors were measured between the proximal incisal contact area positions of the clinical crowns.

Examiner reliability was verified by repeating all measurements on 38 randomly selected records 1 month after the original measurements. Paired *t*-test analysis was used to compare the original and repeated data. None of the measurements showed any statistical difference (*p*=0.05) between the original and repeated values.

## 3. Results

**Figure 1:**
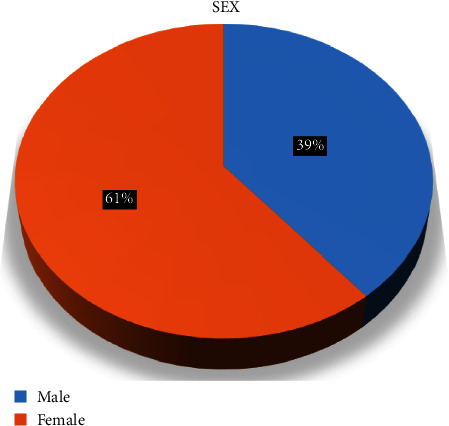
Distribution of the study population according to sex.

**Figure 2:**
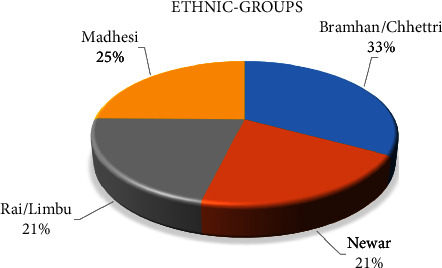
Distribution of the study population according to ethnicity.

A total of 210 subjects of the age group 18–25 years were included in the study. Females were greater in number than males (Figure 1), and the study population included four ethnic groups (Figure 2): Bramhan/Chhetri, Madhesi, Rai/Limbu, and Newar.

Mean GZP of each upper anterior tooth was distally located with respect to VBM; that of CI was more distally placed than LI and C (Table 1). Relative GZL of LI was about 0.40 mm below the reference line (Table 2).

Mean length and mean widths of the clinical crown of the upper anterior teeth were also noted as shown in Tables 3 and 4, respectively. The central incisor width/height ratio obtained was >80% that means a squarer tooth.

## 4. Discussion

The need for undertaking this study was due to insufficient literature discussing those components of esthetics in the population and increasing awareness of the general public about dento-facial esthetics. Due to the lack of reference (guide) for the esthetic restoration or replacement of upper anterior teeth, this study aimed to observe and describe some of the dento-gingival components of the population. It is important to consider tooth proportions and gingival shape and relations when dealing with anterior teeth. The ideal maxillary central incisor width/height ratio is approximately 80% [19] whereas, in this study, the ratio obtained was >80% that means a squarer tooth. With respect to other variables, it was found that the mean GZP of each upper anterior tooth was distally located with respect to VBM; that of CI was more distally placed than LI and C. Mean relative GZL of LI was about 0.40–0.41 mm below the reference line.

Results of the present study is similar to that of a study in which mean GZP from the VBM of CI was 1mm; LI was 0.4mm; in 97.5% of canine population, it was centralizedalong the long axis, and the GZLs of the LIs were more coronal by approximately 1mm[11].

In another similar study, maxillary anterior teeth displayed distally located GZP from VBM, with mean average of 1 mm in the CIs, 0.4 mm in the LIs, and 0.2 mm in the Cs; males and females presented no statistically significant differences; GZL showed statistically significant differences between right and left teeth in males and females and revealed positive values in all the sites (100%) [12].

A similar clinical study on gingival zenith has found the gingival zenith of the Cs apical to the gingival zenith of the incisors and the gingival zenith of the LIs below or on (17%) the gingival line when the head is oriented on the axis orbital plane. Asymmetry was seen when the two sides were compared [10].

It has been seen in a study that the highest rated smiles had the CIs gingival margin matched or 0.5 mm below the line of the Cs gingival margin, and the central-to-lateral incisal step was 1.0 to 1.5 mm. When the CIs gingival margin was 1.0 mm above or 1.5 mm below the Cs gingival margin and no step between the centrals and laterals or a 2.5-mm step, it was considered the worst smile [8].

In a study of gingival zenith position in different facial forms, the mean distance of GZP in relation to VBM of maxillary incisors was 1.06 mm, 1.12 mm, 1.04 mm, and 1.04 mm in oval, square, square tapered, and tapered face types, respectively, and there was a statistically significant difference within the four face types but, no statistical difference in the contralateral comparisons thus, emphasizing bilateral symmetry [13].

Similarly, a study evaluating the GZP and GZL in maxillary anterior teeth in different age groups and genders found that the GZP was distal in 54.68% and 78.12% of the CIs for males and females in Group I, while in Group II it was 65.62% and 75.00%, respectively [17]. The majority of LIs and Cs had a coincidence of the GZP and VBM. The GZL was found to be at an apical position with reference to the GZP of LIs. Distal GZP was observed for CIs, while the GZP coincided with the VBM for LIs and Cs. The GZL was apically placed in relation to the GZP of LIs [17].

One of such studies in Nepal found that, in male, the GZP for right CI, LI, and C was 1.05 mm, 0.57 mm, and 0.14 mm, respectively, and that for the left was 1.02 mm, 0.53 mm, and 0.15 mm, respectively. Similarly, in female, the GZP for the right side was 0.99 mm, 0.48 mm, and 0.15 mm, respectively, and that for right and left lateral incisors was 0.74 mm and 0.71 mm, respectively, whereas, in female, it was 0.76 mm and 0.72 mm, respectively [18].

Thus, some studies in the literature show that the gingival zenith is located distal to the long axis of the maxillary CIs and Cs, whereas it is at the midline of LIs. However, some other studies have shown that LIs can show a deviation of the gingival zenith from the midline and Cs can have coincidence of the GZP and VBM.

It has been said that the gingival shape of the mandibular incisors and the maxillary LIs should exhibit a symmetrical half-oval or half-circular shape whereas the maxillary CIs and Cs should exhibit an elliptical shape. Thus, the gingival zenith (the most apical point of the gingival tissue) is located distal to the longitudinal axis of the maxillary centrals and canines. The gingival zenith of the maxillary laterals and mandibular incisors should coincide with their longitudinal axis [19].

Some consider that Cs gingival margin must coincide with CIs gingival margin and LIs gingival margin must be slightly below the line [9]. Ideal width-height (W/H) ratio (75 to 85% ratios) and symmetry of CIs must be achieved for esthetic outcome [1, 2, 9, 19]. If it is about 75%, the tooth will have a longer pattern, whereas if near 85%, the tooth will have a wider pattern. The ranges of height and width are important to note so that it will be evident what parameter is at fault causing disproportionality of a tooth and need correction. The CIs must be the dominant teeth in the smile, and they must display pleasing proportions [1]. The proportions of the CIs must be esthetically and mathematically correct [5]. There are various guidelines about tooth proportions in an esthetically pleasing smile [1]. Here, it is not the actual size, but the perceived size that these proportions are based on when viewed from the facial aspect.

The concept of beauty has been connected with harmony and harmonic proportions. These proportions are in progression like the CI is 1.618 times larger than the LI, and the LI is 1.618 times larger than the visible part of the C seen from the vertical axis [7]. But, in a study evaluating the width-to-width and width-to-length proportions of maxillary incisors, no golden proportions and standards were detected [15].

Another study also concluded that the golden proportion of maxillary anterior teeth did not exist in the different morphological facial types and was not affected by gender and morphological facial form [6]. The rigidity of this mathematical formula (golden proportion) and the many variables among patients have raised doubts regarding the reliability of this principle. So, this study did not attempt to derive any such proportions (dependent on visual perception) but has simply measured and presented the various dimensions of the teeth which can give an idea of various proportions also.

Limitations of the study are that this study did not attempt to compare various means, and the dimensions of Cs have not been mentioned because, in most of the cases, some degree of attrition of canine tip was seen.

## 5. Conclusion

- Mean GZP of the right CI was 0.49 ± 0.54 mm, of the left CI was 0.42 ± 0.43 mm, of right LI was 0.20 ± 0.34 mm, of left LI was 0.04 ± 0.35 mm, of right C (Canine) was 0.023 ± 0.38 mm, and of left C was 0.07 ± 0.38 mm
- Mean relative GZL of LI was about 0.40–0.41 mm below the reference line
- Mean height of the right CI was 9.34 ± 0.93 mm, and that of the left CI was 9.31 ± 0.87 mm; of the right LI was 7.91 ± 0.98 mm, and of the left LI was 7.92 ± 0.91 mm
- Mean width of the right CI was 8.34 ± 0.57 mm, and that of the left CI was 8.38 ± 0.48 mm; that of the right LI was 6.62 ± 0.58 mm, and that of the left LI was 6.66 ± 0.53 mm

Thus, the mean GZP of each upper anterior tooth was distally located with respect to VBM; that of CI was more distally placed than LI and C. Relative GZL of LI was below the reference line. The central incisor width/height ratio obtained was >80% that means a squarer tooth. Thus, a reference for the population, which can be used as a guide in the treatment of patients with esthetic concerns, was established.

## Figures and Tables

**Table 1:** GZP of upper anterior teeth with respect to VBM of each tooth.

Tooth	Right (in mm)			Left (in mm)		
	Min.	Max.	Mean ± SD	Min.	Max.	Mean ± SD
Central incisor (CI)	−0.97	1.96	0.49 ± 0.54	−0.43	1.33	0.42 ± 0.43
Lateral incisor (LI)	−0.55	1.67	0.20 ± 0.34	−1.02	0.90	0.04 ± 0.35
Canine (C)	−0.87	1.68	0.023 ± 0.38	−0.86	1.44	0.07 ± 0.38

−: “mesial” positioning of the gingival zenith with respect to VBM.

**Table 2:** Relative GZL of LIs.

Right (in mm)			Left (in mm)		
Min.	Max.	Mean ± SD	Min.	Max.	Mean ± SD
−0.40	1.58	0.40 ± 0.40	−0.54	1.57	0.41 ± 0.42

−: “above” the reference line.

**Table 3:** Heights (measured along VBM) of clinical crowns of upper incisors.

Tooth	Right (in mm)			Left (in mm)		
	Min.	Max.	Mean ± SD	Min.	Max.	Mean ± SD
CI	7.70	11.34	9.34 ± 0.93	7.65	11.25	9.31 ± 0.87
LI	5.87	10.25	7.91 ± 0.98	5.87	10.32	7.92 ± 0.91
LI/CI ratio	0.66	0.99	0.84 ± 0.084	0.65	0.99	0.84 ± 0.083

**Table 4:** Widths (measured between the proximal incisal contact area positions) of clinical crowns of upper incisors.

Tooth	Right (in mm)			Left (in mm)		
	Min.	Max.	Mean ± SD	Min.	Max.	Mean ± SD
CI	6.67	9.64	8.34 ± 0.57	7.23	9.45	8.38 ± 0.48
LI	4.98	7.62	6.62 ± 0.58	5.41	7.61	6.66 ± 0.53
LI/CI ratio	0.64	0.96	0.79 ± 0.066	0.65	0.91	0.79 ± 0.059

## Data Availability

The data used to support the findings of this study are included within the article.
